# T Cells and Pathogenesis of Hantavirus Cardiopulmonary Syndrome and Hemorrhagic Fever with Renal Syndrome

**DOI:** 10.3390/v3071059

**Published:** 2011-07-06

**Authors:** Masanori Terajima, Francis A. Ennis

**Affiliations:** Center for Infectious Disease and Vaccine Research, Division of Infectious Diseases and Immunology, Department of Medicine, University of Massachusetts Medical School, Worcester, MA 01655, USA; E-Mail: Francis.Ennis@umassmed.edu

**Keywords:** hantavirus, hantavirus cardiopulmonary syndrome, hemorrhagic fever with renal syndrome, immunopathogenesis, CD8^+^ T cell, regulatory T cell, endothelial cell

## Abstract

We previously hypothesized that increased capillary permeability observed in both hantavirus cardiopulmonary syndrome (HCPS) and hemorrhagic fever with renal syndrome (HFRS) may be caused by hantavirus-specific cytotoxic T cells attacking endothelial cells presenting viral antigens on their surface based on clinical observations and *in vitro* experiments. In HCPS, hantavirus-specific T cell responses positively correlated with disease severity. In HFRS, in one report, contrary to HCPS, T cell responses negatively correlated with disease severity, but in another report the number of regulatory T cells, which are thought to suppress T cell responses, negatively correlated with disease severity. In rat experiments, in which hantavirus causes persistent infection, depletion of regulatory T cells helped infected rats clear virus without inducing immunopathology. These seemingly contradictory findings may suggest delicate balance in T cell responses between protection and immunopathogenesis. Both too strong and too weak T cell responses may lead to severe disease. It is important to clarify the role of T cells in these diseases for better treatment (whether to suppress T cell functions) and protection (vaccine design) which may need to take into account viral factors and the influence of HLA on T cell responses.

## 1. Hantavirus Cardiopulmonary Syndrome and Hemorrhagic Fever with Renal Syndrome

Hantaviruses are RNA viruses possessing a segmented negative-stranded RNA genome belonging to the family *Bunyaviridae*, genus *Hantavirus* [1], and are conventionally divided into the Old World and the New World hantaviruses based on the geographic regions where they occur [2]. The Old World hantaviruses, including Hantaan virus (HTNV), Seoul virus (SEOV), Dobrava virus, and Puumala viruses (PUUV), which are seen throughout Europe and Asia, cause a human disease known as hemorrhagic fever with renal syndrome (HFRS), which is clinically characterized by non-specific flu-like symptoms followed by thrombocytopenia, and a capillary leak syndrome with hemoconcentration. In severe cases renal failure and shock can develop [1,3]. Mortality rates vary from less than 1% to 15%, depending on the individual virus [1,2,4]. The New World hantaviruses include Sin Nombre virus (SNV) and Andes virus (ANDV) seen in the Americas [1,2,4], which were recognized as a cause of disease when the first outbreak of hantavirus cardiopulmonary syndrome (HCPS, it was initially named hantavirus pulmonary syndrome (HPS), which is still being widely used in literature) occurred in the southwestern United States in 1993 (a history of the discovery of the hantaviruses are described in a review by Hjelle and Torres-Pérez in this special issue “Pathogenesis of Emerging and Re-Emerging RNA Viruses” [2]). HCPS shares many characteristics with HFRS, including thrombocytopenia and a capillary leak syndrome except for its target organ. The pathology seen with Old World hantaviruses focuses on the kidney, but the major target organ for the New World hantaviruses is the lung, although there are recent reports of PUUV infection which met the HCPS case definition [5] and renal sequelae observed in HCPS cases [6]. HCPS cases also progress to a severe degree more frequently than do HFRS cases. The latest mortality rate of HCPS in the US is 36% [7].

The molecular characteristics of hantaviruses, and clinical and pathological descriptions of the HCPS and HFRS are also reviewed in this special issue [2].

## 2. Involvement of T Cells in the Pathological Changes

Pathological changes in HCPS and HFRS are characterized by an increased permeability in microvascular beds of affected organs, the kidney in HFRS and the lung in HCPS, and endothelial cells are considered to be the primary targets of hantavirus infection [3,8–11]. *In vitro* hantavirus infection alone, however, did not induce visible cytopathic effects in cultured human endothelial cells [12–15]. Hantavirus infection also induced little or no increase in capillary permeability of an infected endothelial cell monolayer [15–18]. Because direct effects of hantavirus infection on endothelial functions were recently reviewed by Mackow and Gavrilovskaya [19], in this review we will focus on immune-mediated mechanisms of the HCPS and HFRS pathogenesis.

There are observations suggesting immune-mediated mechanisms in the pathogenesis of the HCPS and HFRS (previously published reviews discussing immune mechanisms in hantavirus pathogenesis by us and others are [3,20–25]). (1) In HCPS, large immunoblasts were seen in the circulation at the onset of pulmonary edema and shock [26] and in necropsy lung tissues [10,27], and in HFRS T cell activation in the acute phase was also reported [28,29]; (2) Although the levels of viremia on admission correlated with disease severity in HCPS cases [30,31], viremia was cleared soon after the onset of lung edema, but the disease continued to progress [30]; (3) We showed the presence of high levels of cytokine producing cells in lung tissues from patients with fatal HCPS [32]. The cytokines detected include tumor necrosis factor (TNF)-α, interleukin (IL)-2, IL-6 and interferon (IFN)-γ, which are produced by T cells and may mediate capillary leakage. Exposure to high doses of TNF-α *in vivo* is known to induce shock, capillary leakage, pulmonary edema, and mortality [33], and therapy with high doses of IL-2 causes an increase in vascular permeability [34,35]. Also in nephropathia epidemica (NE), a milder form of HFRS caused by PUUV, lymphocyte infiltration, predominantly of CD8^+^ T cells, was observed in the kidney accompanied by TNF-α expression [36], and elevated plasma levels of TNF-α, IL-6, and IL-10 were reported [37]; (4) Genetic linkage analysis, which will be described in the next section also suggest that the involvement of T cells, especially CD8^+^ T cells, in the pathogenesis of the HCPS and HFRS; (5) An *in vitro* transwell permeability assay demonstrated that SNV infection of human endothelial cell line and the virus-specific CD8^+^ T cells increased permeability, but infection or CD8^+^ T cells alone did not [18]. Autopsies performed on patients with fatal HCPS, however, demonstrated that infected lung endothelial cells were not necrotic and the lung architecture appeared to be grossly intact [10,27]. *In vivo* the main mechanism for increased permeability may be the release of cytokines, such as TNF-α, by the virus-specific CD8^+^ T cells rather than direct lysis of endothelial cells. Interestingly HTNV, SEOV and Dobrava virus were found to inhibit TNF-α-induced nuclear factor κ B activation, but PUUV, SNV and ANDV were not [38,39]. Gavrilovskaya *et al.* reported that human pathogenic hantaviruses, such as HTNV, ANDV and New York-1 virus, did increase sensitivity of infected endothelial cells to vascular endothelial growth factor [40], which has permeabilizing effects on endothelium [41]. In the *in vitro* transwell permeability assay we did not perform experiments identifying molecules mediating the increased permeability.

We previously discussed mouse experiments performed in the context of transplant rejection [42,43] in a brief review [25]. Transgenic mice which expressed β-galactosidase (BG) protein in the endothelial cells were used as a model. Infection of these transgenic mice with recombinant vaccinia virus expressing BG protein induced humoral and cellular immune responses against the BG protein (the BG protein was not tolerated!). The infected transgenic mice, however, remained healthy. No damage was observed in endothelial cells expressing BG protein, which were supposed to be a target of the humoral and cellular immune responses. The transgenic mice also remained healthy after primed spleen cells or lymph node cells from immunized wild type mice, which contained T cells reacting to BG protein, were adoptively transferred into them. These series of experiments suggest that in laboratory mice (FVB background) endothelial cells are somehow protected from humoral and cellular immune responses, which is in contrast with transgenic mouse model of influenza virus infection, in which adoptive transfer of hemagglutinin-specific CD8^+^ T cells into transgenic mice expressing hemagglutinin in lung alveolar epithelial cells induced lung injury [44,45]. Activated endothelial cells are known to express programmed death ligand-1 (PD-L1) and PD-L2, which can down-regulate CD8^+^ T cell activation and lysis [46–48]. In the transplant rejection model, the BG protein-specific CD8^+^ T cells may have been inactivated by these molecules expressed on the endothelial cells, or there may not have been enough BG protein-specific CD8^+^ T cells to overcome this protective mechanism. In HCPS and HFRS patients there may be an overwhelming amount of activated CD8^+^ T cells, or the protecting mechanisms may not be functioning properly because of hantavirus infection of endothelial cells.

Recently Björkstrom *et al.* reported rapid expansion and long-term (>60 days) persistence of elevated NK cell numbers in HFRS patients, which may have relevance to immunopathogenesis [49].

## 3. Genetic Susceptibility to HCPS and HFRS

MHC class I and class II molecules bind and present viral peptide fragments to CD8^+^ and CD4^+^ T cells. Different MHC class I and class II molecules present different sets of viral peptides; therefore, CD8^+^ and CD4^+^ T cell responses in individuals carrying different MHC class I and class II molecules, which are encoded by HLA-A, -B and -C genes (MHC class I molecules) and HLA-DR, -DP and -DQ genes, (MHC class II molecules), may be different [50]. In NE caused by PUUV infection, the HLA-B8-DR3 extended haplotype was associated with severe outcome of the disease [51,52] and with higher PCR positivity of the virus in clinical samples (reflecting prolonged or higher levels of viremia) [53], and HLA-B27 was associated with milder disease [54] in Finland. The B8-DRB1*03 haplotype was also significantly higher in pediatric NE patients [55]. In the Chinese Han population the HLA-DRB1*09 was significantly higher in HFRS patients [56]. Interestingly, as Wang *et al.* pointed out [56], the B8-DR3 haplotype is common in the Finnish population, but rare in the Chinese Han population. In contrast, the DRB1*09 is common in the Chinese Han population, but rare in the Finnish population [57,58]. In HCPS caused by SNV in the southwestern US, the HLA-B*3501 allele was associated with increased risk for developing severe HCPS [59,60], while HCPS caused by ANDV in Chile the HLA-B8 was associated with severe disease and the HLA-DRB1*15 was associated with mild disease [61]. In this Chilean study, contrary to the study in the southwestern US, the HLA-B35 allele was found more frequently in patients with mild disease than in patients with severe disease.

There is one report which clearly showed that the difference in the MHC class I molecule alone can influence disease outcome of viral infection [62]. Flatz *et al.* accidentally observed that humanized mice which express the human/mouse-chimeric HLA-A2.1 molecule instead of the murine MHC class I molecules (HHD mice) developed disease similar to severe Lassa fever (Lassa fever [63,64] is caused by a member of arenavirus family, Lassa virus, not by hantaviruses). The wild type C57BL/6 and the HHD mice had the same levels of viremia up to 4 days after infection, but by day 7 the wild type mice cleared the virus without any clinical evidence of disease, while the HHD mice were not able to clear the virus and some (22%) died. The HHD mice share the same genetic background with the C57BL/6 mice except for the MHC class I; therefore, the results suggested that the wild type mouse’s MHC class I-restricted (*i.e.*, H-2D^b^ and H-2K^b^-restricted) CD8^+^ T cell responses are important for controlling the virus infection and the HHD mouse’s class I-restricted (HLA-A2.1-restricted) CD8^+^ T cell responses cannot control the virus.

## 4. Targets of T Cell Responses

Hantaviruses have four proteins encoded by three genomic RNA segments. Nucleocapsid protein (N) is encoded by the S segment. Two envelope glycoproteins, Gn and Gc (formerly called G1 and G2), are proteolytically processed from the glycoprotein precursor encoded by the M segment. RNA-dependent RNA polymerase (L) is encoded by the L segment [11] In humans CD4^+^ and CD8 T^+^ cell epitopes on hantavirus proteins were identified by analyzing peripheral blood mononuclear cells (PBMCs) obtained either from donors who had immune memory to the virus [65–69] or from HCPS or HFRS patients in the acute phase [60,70–72]. Well-characterized epitopes (minimal epitopes and HLA restrictions have been determined, and specific T cells have been detected in or specific T cell lines have been generated from human PBMCs) are listed in Table 1. In HCPS caused by SNV and ANDV, very high frequency of single epitope-specific CD8 T cells were reported by MHC/peptide tetramer staining (G_664–673_ and G_746–755_ for SNV and G_465–473_ for ANDV) [60,69]. These epitopes are on the envelope glycoproteins. In contrast, in HFRS most epitopes identified to date are on the N of Hantaan virues (HTNV) and PUUV. PBMCs from some of HTNV- and PUUV-immune donors showed cytotoxicity against the Gn or Gc after *in vitro* stimulation by the virus, although N were recognized more frequently than Gn or Gc [65,67]. These studies were very limited. Except for one study testing a small number of peptides identified by an MHC class I binding motif search on the L [71], none of these studies tried to detect T cell responses to L or to identify T cell epitopes on the L protein.

Studies analyzing donors who had experienced HCPS or HFRS years ago showed that T cell memory is long-lived [65–67,69], although one report implied the possibility of persistent presence of ANDV antigen after acute infection [69].

## 5. Disease Severity and T Cell Responses—Published Clinical Studies

By analyzing PBMC samples obtained from HCPS patients with MHC/peptide tetramer staining we showed that there were high frequencies of SNV epitope-specific CD8^+^ T cells in the acute phase [60] compared to the frequencies of epitope-specific CD8^+^ T cells in the acute phases of other viral infections, such as human immunodeficiency virus, hepatitis C virus, hepatitis B virus, vaccinia virus and dengue virus, quantitated by MHC/peptide tetramer staining, in which the frequencies were often <1% [73–81]. Acute infectious mononucleosis by Epstein-Barr virus is the only other human viral infection with comparable frequencies of epitope-specific CD8^+^ T cells [82–85]. We also found that the frequencies were significantly higher in eight patients with severe HCPS requiring mechanical ventilation (16.5 ± 5.9% (average ± standard deviation) of CD8^+^ T cells at earliest time points, 18.3 ± 6.6% at peak responses) than in three moderately ill HCPS patients hospitalized but not requiring mechanical ventilation (5.9 ± 3.5% of CD8^+^ T cells at earliest time points, 6.6 ± 2.8% at peak responses) (in the original paper [60], we used the Wilcoxon rank sum test to compare the severe and the moderate cases, and found statistically significant differences both at earliest time points and peak responses), suggesting immunopathological roles of these SNV-specific CD8^+^ T cells.

In HFRS, contrary to the finding in HCPS, Wang *et al.* reported that frequencies of HTNV-specific cells, which were quantitated by ELISPOT assays using overlapping peptides covering the N protein of HTNV, were significantly higher in patients with mild or moderate HFRS than in those with severe or critical HFRS, suggesting protective roles of these HTNV-specific cells [86].

There are several critical methodological differences between these two studies. The HCPS study [60] used HLA-B*3501/peptide tetramers to detect SNV-specific T cells, which enabled us to analyze only patients carrying the HLA-B*3501 allele (risk factor for severe HCPS and relatively common allele among HCPS patients [59]). The tetramers detected CD8^+^ T cells specific to three epitopes (N_131–139_, G_664–673_ and G_746–755_ in Table 1) irrespective of these T cells’ function. CD8^+^ T cells specific to other epitopes could not be analyzed by these tetramer staining. The HFRS study [86] used IFN-γ ELISPOT assays to detect HTNV-specific T cells. PBMCs were stimulated with pools of peptides covering the entire N protein to produce IFN-γ, meaning that only T cells which were specific to the N protein and were able to secrete IFN-γ were detected.

In the study analyzing CD8 T cell memory generation in NE patients caused by PUUV Tuuminen *et al.* found huge difference between frequencies of PUUV N_204–212_-specific T cells quantitated by HLA-A2/N_204–212_ tetramer staining and by IFN-γ ELISPOT assays with the same peptide in the PBMC obtained in the acute phase of the disease (3200 ∼ 19800 N_204–212_-specific cells per million PBMCs by the tetramer staining and <50 ∼ 300 by IFN-γ ELISPOT assays at the presentation to hospital) and during the follow-up the difference became much smaller [87]. Therefore, by the tetramer staining PUUV-specific T cells showed expansion and contraction, but by IFN-γ ELISPOT assays PUUV-specific T cells showed a gradual increase. By the *ex vivo* analysis combining the tetramer staining and intracellular cytokine staining the authors found that on average 15.2% of tetramer-positive cells in the acute phase and 5.1% in the convalescent phase expressed IFN-γ, suggesting that the IFN-γ ELISPOT assays underestimated the frequency of PUUV N_204–212_-specific T cells in the acute phase samples. They also showed that cells isolated during the acute phase were more prone to activation-induced apoptosis than cells isolated during convalescence, which may account for the difference which cannot be explained by the low percentage of IFN-γ-producing cells in the tetramer positive population in the acute phase. A similar scenario may have happened to HTNV-specific T cells in the HFRS study, in which the numbers of HTNV-specific T cells (specific to the N protein) in the acute phase quantitated by IFN-γ ELISPOT assays were in the range of hundreds, not in the thousands [86]. Quantitating HTNV-specific T cells by tetramer staining is likely to clarify if there is early expansion of the specific T cells in the acute phase of HFRS and if there is correlation between the disease severity and the frequencies of the specific T cells. It is also important to quantitate T cells specific to epitopes restricted by other alleles (so far only B*3501/peptide tetramers [60,69] and HLA-A2/peptide tetramers [87] have been used for quantitation).

## 6. Syrian Golden Hamster Model of HCPS

During vaccine studies, ANDV, but not SNV, was found to cause disease very similar to HCPS in Syrian golden hamsters [88,89]. Both viruses infect Syrian golden hamsters, but only ANDV causes viremia [90]. Two New World hantaviruses which are not known to cause diseases in humans, Maporal virus and Choclo virus, were also tested in Syrian golden hamsters [91,92]. While Maporal virus caused a disease pathologically similar to the HCPS-like disease caused by ANDV [91], Choclo virus did not cause any symptoms of the disease. When compared to animals infected with ANDV, the prevalence of antigen-positive endothelial cells in the lungs of Choclo virus-infected animals was similar, but no inflammatory cellular infiltrate or lung edema were observed [92], suggesting that host immune responses are necessary for Syrian golden hamsters to develop the HCPS-like disease.

## 7. Regulatory T Cells in Hantavirus Infection

The natural reservoir of hantaviruses are persistently infected rodents [93]. There are many mechanisms proposed to explain viral persistence and the relative absence of disease in these rodent reservoirs (reviewed in [93,94]). One of them is suppression of proinflamatory and effector T cell activity by regulatory T cells [95,96].

Seoul virus, which causes HFRS, is maintained in Norway rats. Easterbrook *et al.* hypothesized that Seoul virus may exploit regulatory T cell responses to cause persistence in rats [97]. To test their hypothesis, regulatory T cells were inactivated by injecting anti-CD25 monoclonal antibody into rats inoculated with Seoul virus (Seoul virus infection increases CD4^+^CD25^+^FoxP3^+^ regulatory T cells in these rats). Consistent with the hypothesis, inactivation of regulatory T cells reduced the amount of Seoul virus RNA in the lung (target organ of the Seoul virus infection in rats) and saliva. Absence of regulatory T cells may result in uncontrolled effector T cell activity and causes tissue damage through immunopathological mechanisms. In this experimental setting, however, the average amount of hemorrhage and edema in the lung was not increased in rats whose regulatory T cells were inactivated by the anti-CD25 antibody injection [97]. There is a possibility that, because activated T cells also express CD25, anti-CD25 antibody treatment may have inactivated activated effector T cells as well as regulatory T cells and prevented immunopathology. The authors think it unlikely because during acute and persistent Seoul virus infection >90% of CD4^+^CD25^+^ T cells were FoxP3^+^ regulatory T cells.

Abel Borges and Figueiredo speculated that active suppression of immune T regulatory cells is probably involved in HCPS pathogenesis [98]. Zhu *et al.* analyzed regulatory T cells in patients with HFRS [99]. They found that, in contrast to Norway rats infected with Seoul virus [97], the frequency of regulatory T cells identified as CD4^+^CD^25high^ was reduced in the acute phase of HFRS compared to the convalescent phase and levels in healthy controls. In these HFRS patients the frequencies of regulatory T cells inversely correlated with disease severity, suggesting that inefficient control of T cell effecter functions may be responsible for more severe disease, although HNTV-specific T cells were not directly analyzed in this study and identifying regulatory T cells by CD4 and CD25 expression alone in humans may be controversial [100]. It should also be noted that in Norway rats Seoul virus causes persistent infection, not acute infection similar to HFRS.

## 8. Immunopathological Mechanisms

Numerous studies of influenza virus infection (reviewed in [101]) suggest that almost all mediators of pathological changes are also essential for efficient viral clearance, which is likely to be true to hantavirus infection. There are two possible scenarios of immunopathological mechanisms for HCPS and HFRS. (1) T cell responses eliminate virus, but intensive T cell responses may alter endothelial cell function (capillary leakage) by secreting an excessive amount of cytokines. (2) Conversely, if sufficient amount of T cell responses is not induced, virus clearance is delayed leading to prolonged inflammation, which may also alter endothelial cell function by non-T cell-mediated mechanisms (e.g., as mentioned earlier, human pathogenic hantaviruses can increase sensitivity of infected endothelial cells to vascular endothelial growth factor [40]). The HCPS clinical study, in which the frequencies of the virus-specific T cells correlated with disease severity [60], and the HFRS clinical study by Zhu *et al.*, in which decreases in the numbers of regulatory T cells (presumably meaning increases in effector CD8^+^ T cell responses) correlated with disease severity [99], correspond to the first scenario that intensive T cell responses cause the HCPS and HFRS. The other HFRS clinical study by Wang *et al.* reporting that frequencies of virus-specific T cells inversely correlate with disease severity [86] corresponds to the second scenario.

## 9. Conclusions

In both HCPS and HFRS, CD8^+^ T cell responses are essential for elimination of virus-infected cells and viral clearance. Both too strong and too weak T cell responses may lead to severe disease. It is important to clarify the role of T cells in these diseases for better treatment (whether to suppress T cell functions) and protection (vaccine design) which may need to take into account viral factors and the influence of HLA on T cell responses.

## Figures and Tables

**Table 1:** CD8^+^ T cell epitopes identified in hantavirus proteins ^a^.

**Epitope**	**Amino Acid Sequence**	**Virus**	**HLA Restriction**	**Reference**
N131–139	LPIILKALY	Sin Nombre	B*3501	[70]
N234–242	ERIDDFLAA	Sin Nombre	Cw7	[70]
G664–673^b^	TAHGVGIIPM	Sin Nombre	B*3501	[60]
G746–755^b^	YPWQTAKCFF	Sin Nombre	B*3501	[60]
G465–473^c^	LMPDVAHSL	Andes	B*3501	[69]
N12–20	NAHEGQLVI	Hantaan	B51	[65]
N421–429	ISNQEPLKL	Hantaan	A1	[65]
N334–342	ILQDMRNTI	Hantaan	A2.1	[71]
L512–520	ILPSKSLEV	Hantaan	A2.1	[71]
L1273–1281	IMELATAGI	Hantaan	A2.1	[71]
L1736–1744	GLDCARLEI	Hantaan	A2.1	[71]
N173–181	RPKHLYVSM	Puumala	B7/B8^d^	[67]
N204–212	GLFPTQIQV	Puumala	A2.1	[67]
N243–51	ECOFIKPEV	Puumala	B8	[67]
G731–939^b^	HWMDATFNL	Puumala	A24	[66]

a For all epitopes listed, minimal epitopes and HLA restrictions have been determined, and specific T cells have been detected in or specific T cell lines have been generated from human PBMCs;

b Amino acid numbering is based on the sequence of glycoprotein precursor. These epitopes are located on the Gc;

c Amino acid numbering is based on the sequence of glycoprotein precursor. This epitope is located on the Gn;

d Restricted by both HLA-B7 and B8 molecules.
